# The Prelimnary Education of Medical Students

**Published:** 1861-12

**Authors:** 


### Selections.

THE following remarks from the American Medical Times of Oct. 12, upon the "Preliminary education of medical students," applies with equal force to the dental student:

THE PRELIMINARY EDUCATION OF MEDICAL STUDENTS.—There is no literary institution in the United States that does not put every student that seeks to enter its halls to the test of a rigid examination in the elementary branches of learning. If not found proficient, or not to have attained the required standard, he is refused admission, and compelled to turn back and qualify himself for those higher studies, or seek some employment better suited to his talents and acquirements. We are not aware that any one has complained that this system is too rigid, or that it is unjust. No one has even suggested that it were better to allow every student who applies for admission to the classes of our literary institutions to go through a regular course unchallenged and obtain what education he could, urging that thereby he would be a more useful man than he possibly could be uneducated. The position would certainly not be irrational, and might be maintained with a good array of arguments. On the contrary, all interested in the cause of education unite in sustaining the system, and give the best support to the colleges whose examination is most stringent. Nor do the professors in these institutions complain that by being thus careful in guarding the portals to the halls of learning they so effect a diminution of their classes as to endanger the very existence of their respective schools. There exists among them a spirit of emulation, which exhibits itself in efforts to graduate classes well appointed by education to take high rank in subsequent life, rather than in measures to simply swell numbers regardless of their educational qualifications. The true measure of success with them is not the quantity but the quality of the material manufactured.

Should a literary institution be established which admitted whoever chose to apply, without an inquiry into the moral character or preliminary education of the applicant until the completion of the prescribed course, we can have no doubt as to the rank which it would take. It might boast of well filled halls, of overflowing classes, of a long catalogue of patrons, but it would never refer to the educational character of its graduates—the real test of its merits. Such a literary college would soon become a hissing and a byword among the educated, and would speedily sink under the load of infamy which it would call down upon its devoted head by such a prostitution of its powers. It would be repudiated by every honorable and high-minded student, and its diploma would be little better than if issued by the Castle of Indolence. Its testimonial of proficiency in learning would be but a price set upon idleness, incompetency, and demerit.

While our literary institutions exhibit such commendable zeal in behalf of a high standard of education, and consider their chief excellence to rest in the character and not the number of their graduates, our institutions for medical learning pursue a diametrically opposite policy. They esteem the true measure of success to be the number of their graduates, and not the proficiency of these graduates in medical science. Their doors are not only thrown widely open and every one invited to enter, but, in some cases, their servants have been sent out into the highways and byways to compel students to come in that their lecture-rooms might be full. No test questions must be put to such guests, lest they should take it as an insult and attend a neighboring school. The only preliminary examination ever instituted, that we are aware of, was as to the *color* of the student. Some schools have not even the courage to exact the stipulated fee, lest they should give offense and diminish their classes, while nearly all swell their lists with the names of many who are not full students of medicine. Under the title "beneficiaries," many colleges contrive to admit large classes who are totally unfit for the study, and much more the practice of medicine. All colleges agree in waiving an examination into the moral character and qualifications, by preliminary education, of the student, until he has completed the course of three years of study, and an attendance upon two full courses of lectures. And what if he is then found unqualified? Ah! but who ever heard of a medical student after "three years' study and an attendance upon two full courses of lectures, the last of which was in *this institution,*" who was not found qualified? It is too cruel after three years of study, and especially after having attended the last full course of lectures (and paid his fees) at "*this* institution," to tell him flatly that he is not qualified to practice medicine. And what an amount of assurance on the part of a Faculty would it not require to do their whole duty in many instances, and candidly inform the candidate that he has altogether mistaken his calling; that he was never qualified, either by natural or acquired mental force, or by early education, for the profession of medicine; that, in a word, he has wasted both time and money in his present pursuit, and must now, after fulfilling all the required terms for graduation, except passing a "satisfactory examination," give up the course of life which he and his friends had marked out for him, and seek some more congenial avocation. No Medical Faculty, however high-minded, would have the moral courage to take from a student his time and his hard-earned fees, and then deliberately tell him the truth in regard to his qualifications. Many conscientious professors, anxious to do their duty to the profession, and yet sympathizing with the student, whose future life trembles in the balance, are annually put on the rack. With many a doubt, and much hesitation, they at last yield to the force of that policy which makes it too late to deny the student, when he is first put to the test of a rigid examination. Thus many of our best schools are betrayed into granting their diplomas to graduates who not only disgrace them, but who hang like a millstone around the neck of legitimate medicine. It is from this class that quackery, to the everlasting shame of the medical educating bodies, gains its recruits. Nor can we expect better things until a radical and complete reform is made in our system of medical education. And that reform is suggested by the practice of literary institutions of examining candidates on their first application for admission to the course of instruction. No consideration, other than the desire to do justice primarily to the student, and secondarily to the school, could then influence the judgment of the examiner. If the applicant were disqualified by want of natural abilities to acquire a proper knowledge of medicine, he would unhesitatingly inform him, and thus doubtless would persuade him to abandon a pursuit for which he was not adapted. If he were but partially qualified by preliminary education, we would advise him to establish first the basis upon which he was to build. Thus the profession would be saved the infliction of membership with the incompetent and uneducated graduates which now annually swell its ranks.

Although the institution of this reform, and its practical fulfilment, rests with the medical schools, yet the experience of the past has taught us that the impelling power is with the great body of the profession. While the false idea of merit obtains among colleges that the size, and not the educational excellence of the graduating classes, is to be regarded, no reform can be expected. The profession at large must destroy this false and pernicious system, create a new standard, and bring the schools to its test. We are glad to notice that this first step in the reform of our system of medical education is already attracting much and deserved attention. In the Medical Society of the State of New York, Prof. HOWARD TOWNSEND has brought it prominently forward in an able report; in the Indiana State Medical Society it has also been urged in an elaborate report; and at the last meeting of the General Council of Medical Education, England, the report upon medical education contains, as its first proposition, the examination of students as to their having acquired the prescribed preliminary education.

We are but too well aware that the subject of medical education is a trite and hackneyed theme. It has been discussed in all our societies, local and general, until it would seem that no new aspect of the subject could be presented; certainly until all have become thoroughly tired of listening to the reports and debates to which it invariably gives rise. Few of our readers will, we fear, even have the courage to glance down the column bearing its caption, to learn what may perchance be the particular topic discussed, and the arguments brought forward. Nevertheless, it is a subject with which no one should become weary, as it is of vital importance to his own respectability, and the social position of his profession. Certainly no one who studies to promote the best interests of that profession will ever become indifferent to the character of his associates as influenced by education. We believe, therefore, to a certain number the subject of medical education has still much interest, and that suggestions in regard to it will not fall on ears indifferent to their importance.

